# Release performance and kinetic behavior of volatile products from controlled pressure pyrolysis of oil shale in nitrogen atmosphere

**DOI:** 10.1038/s41598-023-37459-5

**Published:** 2023-07-01

**Authors:** Shuai Zhao, Jianzheng Su, Junwen Wu

**Affiliations:** 1 State Key Laboratory of Shale Oil and Gas Enrichment Mechanisms and Effective Development, Beijing, 1100083 China; 2 State Center Research and Development of Oil Shale Exploitation, Beijing, 1100083 China; 3 School of Mines, China University of Mining and Technology, Xuzhou, 221116 China

**Keywords:** Petrology, Engineering, Chemical engineering

## Abstract

The gas injection parameters such as temperature, pressure and duration during the in-situ pyrolysis of oil shale are important factors that affect the pore evolution and product release characteristics of oil shale. This paper takes Huadian oil shale as a sample, uses pressurized thermogravimetry and pressurized fluidized bed experimental device to explore the influence of temperature, pressure and time on the evolution of pore structure under high-pressure nitrogen injection conditions, and analyzes the influence mechanism of pore structure evolution on the release and kinetic behavior of volatile products. The results show that in the range of 623–673 K, the effective oil recovery of oil shale pyrolysis under high pressure increases from 30.5 to 96.0% with the extension of temperature and pyrolysis time, and the average activation energy is 346.8 kJ/mol, which is higher than the activation energy of 306.6 kJ/mol under normal pressure pyrolysis. Under high pressure, the release process of volatile products is inhibited, resulting in the intensification of the secondary reaction of products and the reduction of olefin content. In addition, the primary pores of kerogen are prone to coking reaction and collapse of plastic structure, so that some large pores become microporous structure, and the average pore size and specific surface area are reduced.

## Introduction

Oil is an important guarantee resource for a country's economic development. According to the BP World Energy Statistics Yearbook released by BP oil company in 2022, the world oil consumption will increase by 5.3 million barrels per day in 2021, including 1.3 million barrels per day in China^1^. China is a country with poor oil. In 2021, the dependence of crude oil import on foreign countries was as high as 72%. There is still the risk of oil resources instability^2^. Promoting the exploitation of unconventional energy is one of China's means to cope with the shortage of oil resources^3^. Oil shale is a kind of fine-grained sedimentary rock rich in kerogen, with an oil content of 3.5–30%. After being heated and cracked, shale oil and shale gas can be released^4^. The oil shale in-situ pyrolysis process is based on the hydraulic fracturing to promote the connection of the reservoir, and inject the heat-carrying medium such as nitrogen, then the medium and the oil shale reservoir will conduct convection heat transfer to realize the pyrolysis of oil shale^5^. Due to the different burial depths of oil shale reservoirs, the pressure of injecting heat-carrying media is also different.

In recent years, the research on oil shale pyrolysis has focused on kinetics^6,7^, thermodynamics^8,9^, effective recovery factor^10–12^, pyrolysis method^13,14^, pyrolysis temperature^15,16^, etc. Baruah et al. studied the kinetics and the effect of pressure on product yield of oil shale pyrolysis under non-isothermal and high-pressure conditions. They believe that with the increase of pyrolysis pressure, the secondary reaction of oil vapor will occur, affecting the oil composition and reducing the oil yield^17^. Zhang et al. tested the permeability change characteristics of oil shale pyrolysis in nitrogen atmosphere with axial pressure of 3.75 MPa and confining pressure of 4.5 MPa. They found that under the joint action of gas slippage effect, adsorption effect and effective stress, permeability almost linearly decreases with the increase of pore pressure^18^. Geng et al., using microscopic CT, mercury intrusion and SEM tests to carry out fine characterization of the oil shale pyrolysis process, under the experimental conditions of 5–15 MPa. The results showed that the growth of external pressure and the effect of thermal stress jointly promoted the expansion and fracture of the original and new pore fracture groups, resulting in an increase in the total pore volume and the number of fractures^19^. Liu et al. studied the anhydrous pyrolysis of oil shale under high pressure, and found that high pressure can promote the development of micropores and fine-medium pores in highly mature shale^20^. Li et al. reported that under vacuum conditions, the yield of tar increased, and the aromatic content of tar decreased. They believed that pyrolysis under reduced pressure could obtain higher yield and better quality, which would be beneficial to improve oil production^21^.

As early as 2003, when Roberts et al.^22^ and Wall et al.^23^ conducted pressure pyrolysis of coal, they found that the increase of pressure would change the pore structure of semi-coke in the pyrolysis products, resulting in an increase in the amount of volatile matter released, which affected the gasification activity of coal char. This is consistent with the demonstration by Liu et al.^24^ and Wu et al.^25^ through experiments. In addition, under the conditions of subcritical water^26,27^, water vapor^13,28^ and nitrogen^29,30^, the evolution of pore structure of oil shale has been studied, but under the conditions of high pressure, the influence mechanism of pore structure evolution on the coking and product release behavior of shale oil is less studied. To sum up, the effects of environmental atmosphere, temperature and pressure on oil shale pyrolysis have been extensively studied. However, most of the research focuses on the detailed description of product components. And few studies reveal the effect of pyrolysis pressure on the release behavior of volatile products from the perspective of kinetics based on the actual burial conditions. The sedimentary environment of oil shale is similar to coal^31,32^. In view of this, the release characteristics of volatile products and the kinetic behavior of oil shale pyrolysis under high pressure were investigated by using a pressurized thermogravimetric device and a pressurized fluidized bed experimental system at a nitrogen injection pressure of 7.8 MPa. The composition of pyrolysis products and the pore size distribution and specific surface area of residue were quantitatively analyzed, to improve the effective recovery of oil shale in-situ pyrolysis under high pressure.

## Materials and methods

### Experimental materials

The oil shale with commercial use value in China is mainly located in Fushun (Liaoning Province), Maoming (Guangdong Province), Huadian, Nong'an (Jilin Province) and Longkou (Shandong Province)^33,34^. The oil content of oil shale from different origins is different.The density of Huadian oil shale is about 1.40–1.80 g/cm^3^, and it is mainly composed of quartz, kaolinite, montmorillonite, muscovite, calcite, dolomite, kerogen and other minerals. The higher the density class, the more inorganic minerals are contained in the sample, and the lower the content of kerogen is^35,36^.

The surface of the oil shale sample used in the experiment is brown and dark brown, which is from the Huadian mining area in Jilin Province. Before the experiment, the oil shale sample is broken into small pieces of 1.2–1.5 cm^3^, mixed evenly, and then place the oil shale pieces in an oven at 333 K to dry for 12 h. After the quality of the sample does not change, it shall be stored in sealed bags. Conduct proximate analysis, Fisher oil content test and element analysis on dried powder samples, and the results are shown in Tables 1, 2, 3.

**Table 1:** Proximate analysis of Huadian oil shale.

Attribute	Moisture wt%	Ash wt%	Volatiles wt%	Fixed carbon wt%
Sample1	5.03	52.81	40.02	2.14
Sample 2	5.18	53.06	39.44	2.32

**Table 2:** Fisher analysis of Huadian oil shale.

Attribute	Oil wt%	Water wt%	Residue wt%	Gas wt%
Sample 1	20.31	5.67	63.32	10.70
Sample 2	19.19	5.49	64.73	10.59

**Table 3:** Element analysis of Huadian oil shale.

Attribute	H wt%	C wt%	N wt%	S wt%
Sample 1	4.42	30.91	0.67	2.174
Sample 2	4.16	29.69	0.63	2.161

The proximate analysis results show that the free water content in the dried Huadian oil shale is low, only about 5%. The average content of volatile matter is about 40%, and the average content of ash is 50%. Fisher analysis shows that the oil content of Huadian oil shale is about 19–20%. The gas content is between 10 and 11%, and the mass of semi-coke after pyrolysis is about 64%. In addition, element analysis shows that the main chemical composition of Huadian oil shale is carbon, which also contains a certain amount of hydrogen and sulfur, as well as a small amount of nitrogen.

### Experimental process of pressurized fluidized bed

**Figure 1:**
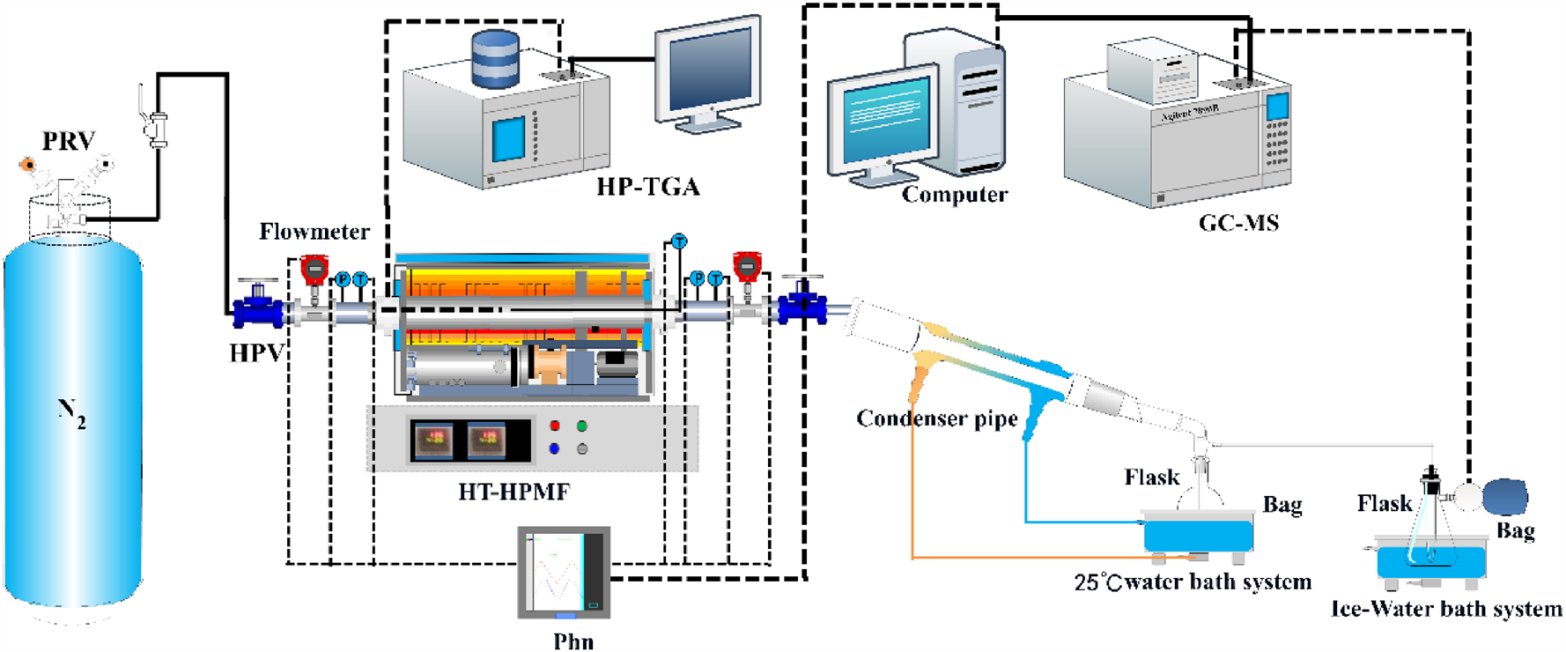
The schematic diagram of the high-pressure pyrolysis setup.

The experimental system is shown in Fig. 1. The specific experimental process is as follows: Firstly, in order to eliminate the influence of air in the fluidized bed on the experimental atmosphere, high-pressure nitrogen gas is used as the environmental atmosphere and purging gas, and the fluidized bed is purged for 2 min. Secondly, Huadian oil shale samples were placed in a high-pressure fluidized bed, the pressure of nitrogen was slowly increased to the experimental pressure, and the high-pressure fluidized bed was heated after the heating rate was set. Thirdly, the pyrolysis oil and gas products enter the condensation pipeline through the displacement effect of high-pressure nitrogen, which includes a two-stage condensation system. The first stage condensation system uses room temperature (approximately 298 K) water, while the second stage condensation system uses an ice water mixture. After two-stage condensation of oil and gas products, shale oil and shale gas are collected respectively. Finally, the composition of the product was analyzed by gas chromatography and mass spectrometry (GC–MS).

In order to eliminate the sample that cannot be completely pyrolysed due to uneven heating, we have made the following modifications to the high-pressure fluidized bed: (1) Use the tube furnace as the heating source, install three heating tubes with power of 700 W on the upper and lower wall of the tube furnace, and the angle between each heating tube is 60°; (2) The high-pressure fluidized bed is placed inside the tubular furnace, which controls the temperature and holding time of the whole fluidized bed; (3) the nitrogen in the gas cylinder directly leads to the fluidized bed through the high-pressure throttle valve.

### kinetic calculation method

**Figure 2:**
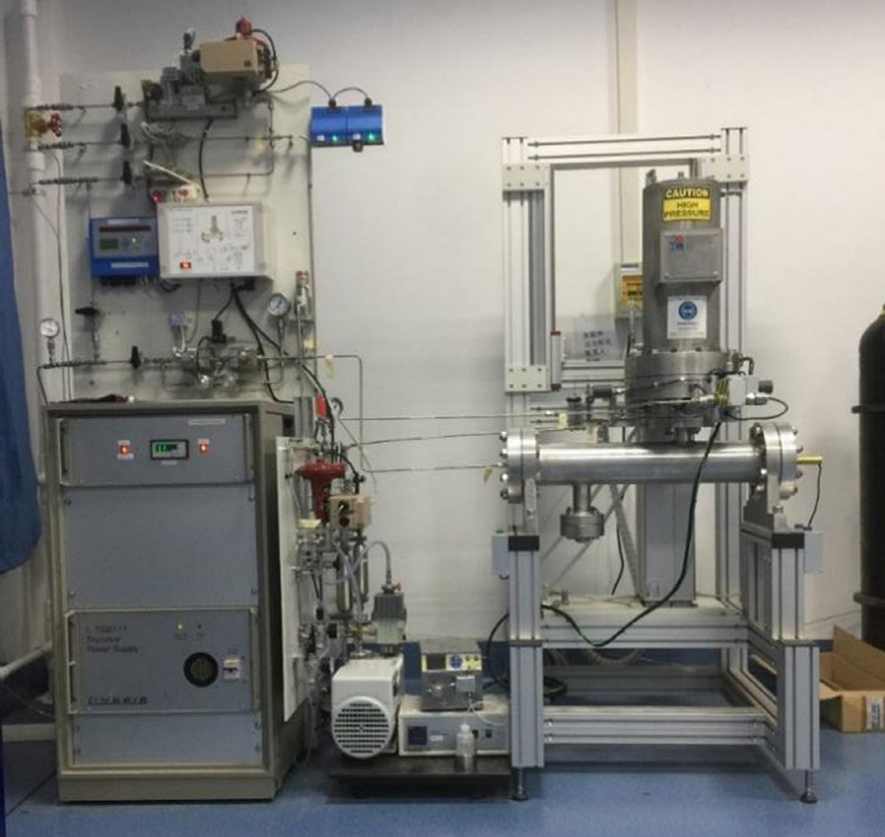
LINSEIS high pressure STA.

The high pressure (7.8 MPa) thermogravimetric test of Huadian oil shale was carried out by LINSEIS High Pressure STA produced in Germany, as shown in Fig. 2. The thermogravimetric test under standard atmospheric pressure was carried out by STA449F3 synchronous thermal analyzer produced by Netzsch in Germany. In order to avoid the calculation error caused by the choice of kinetic calculation method, this paper adopts two different differential calculation methods, KAS method and Friedman method, as the calculation method of apparent activation energy.

Thermogravimetric data show that the pyrolysis of Huadian oil shale under nitrogen atmosphere is divided into three distinct stages, no matter under standard atmospheric pressure or high pressure, and with the increase of heating rate, the pyrolysis range of oil shale moves to high temperature zone^37^, which is more obvious under high pressure.

**Figure 3:**
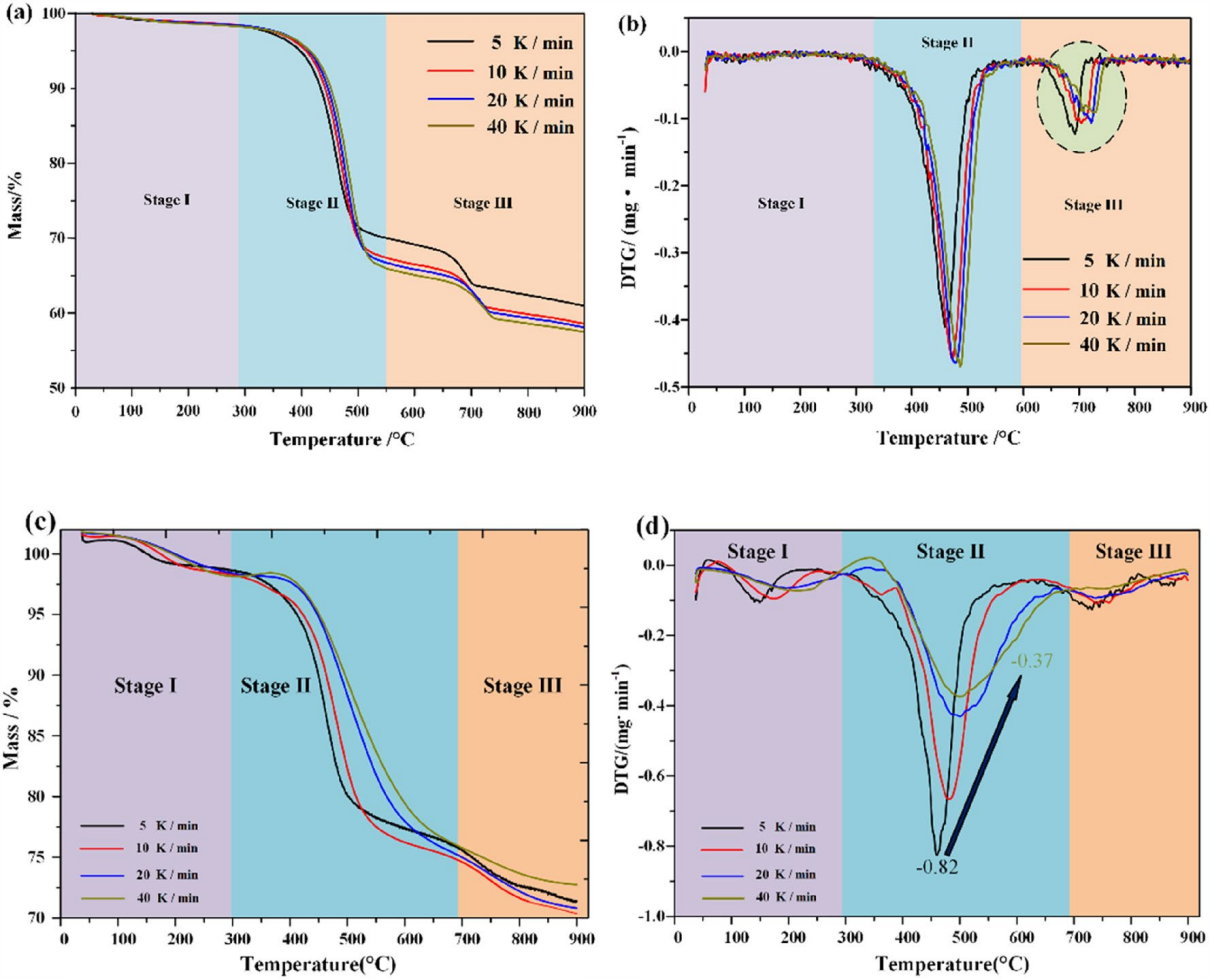
Thermogravimetric diagram of Huadian oil shale under standard atmospheric pressure and high pressure. (**a**) Standard atmospheric pressure TG. (**b**) Standard atmospheric pressure DTG. (**c**) High pressure TG. (**d**) High pressure DTG.

As shown in Fig. 3, the second stage of oil shale pyrolysis is widened under high pressure. This is because the oil and gas products after kerogen pyrolysis need to accumulate more pressure in the pores to break through the constraint of the minimum principal stress, and then release the oil and gas products. In addition, when the heating rate increases from 5 to 40 K/min, the maximum product release rate of oil shale pyrolysis slightly increases under normal pressure. However, the maximum product release rate of oil shale pyrolysis under high pressure decreased from 0.82 to 0.37 mg/min.

#### KAS calculation method

Formulas (1) and (2) are the KAS calculation method^38^:

1 $$\mathrm{\alpha }=\frac{{m}_{0}-m}{{m}_{0}-{m}_{\infty }},$$

2 $$\mathrm{ln}\frac{\beta }{{T}^{2}}=\mathrm{ln}\left[\frac{AR}{{E}_{a}}\right]-\frac{{E}_{a}}{RT},$$

where α is the conversion rate of oil shale pyrolysis, %; m_0_ is the initial mass of oil shale sample, mg; m-mass of sample at T K, mg; m_ꝏ_ is the final mass of the sample, mg; β is the heating rate, K/min; T is the reaction temperature, K; R is the gas constant, 8.314 J/mol; G(ɑ) is the the integral form of the most probable mechanism function; A is the the pre-factor, s^−1^; E_ɑ_ is the apparent activation energy, kJ/mol.

**Figure 4:**
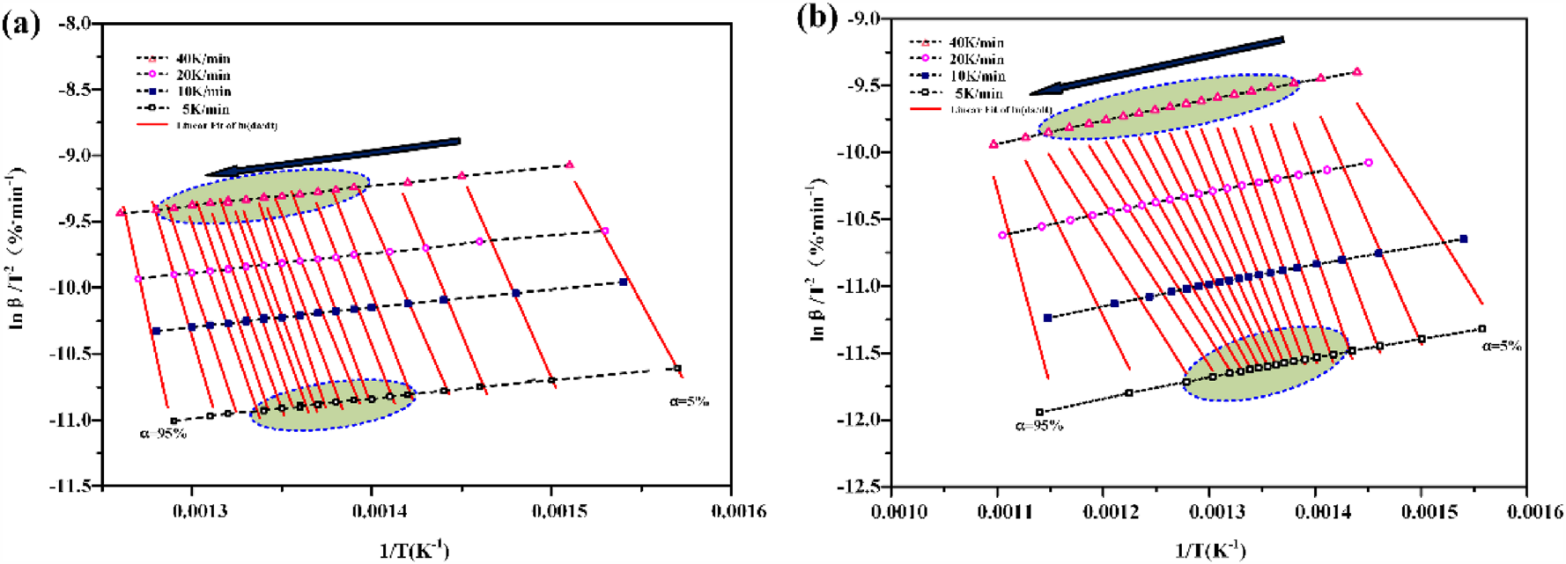
The curve of $\mathrm{ln}\frac{\beta }{{T}^{2}}-$(− 1/T) (**a**) atmospheric thermogravimetry (**b**) high pressure thermogravimetry.

It can be seen from the formula (2) that $\mathrm{ln}\frac{\beta }{{T}^{2}}$ is a linear function of − 1/T, as shown in Fig. 4. The least square method is used to fit the data to obtain the slope k. K = E_ɑ_/RT, then the apparent activation energy E_ɑ_ at the corresponding conversion rate can be obtained. Calculate the corresponding pre-index factor A according to the graph intercept.

#### Friedman calculation method

According to Arrhenius law, the reaction rate can be expressed as^39^:

3 $$\frac{d\alpha }{dt}=\frac{A}{\beta }exp\left(-\frac{{E}_{b}}{RT}\right),$$

where t is time, s; dα/dt is the conversion rate, %.

Take logarithms on both sides at the same time,

4 $$ln\frac{d\alpha }{dt}=\mathrm{ln}\left(A-\beta \right)-\frac{{E}_{b}}{RT}.$$

**Figure 5:**
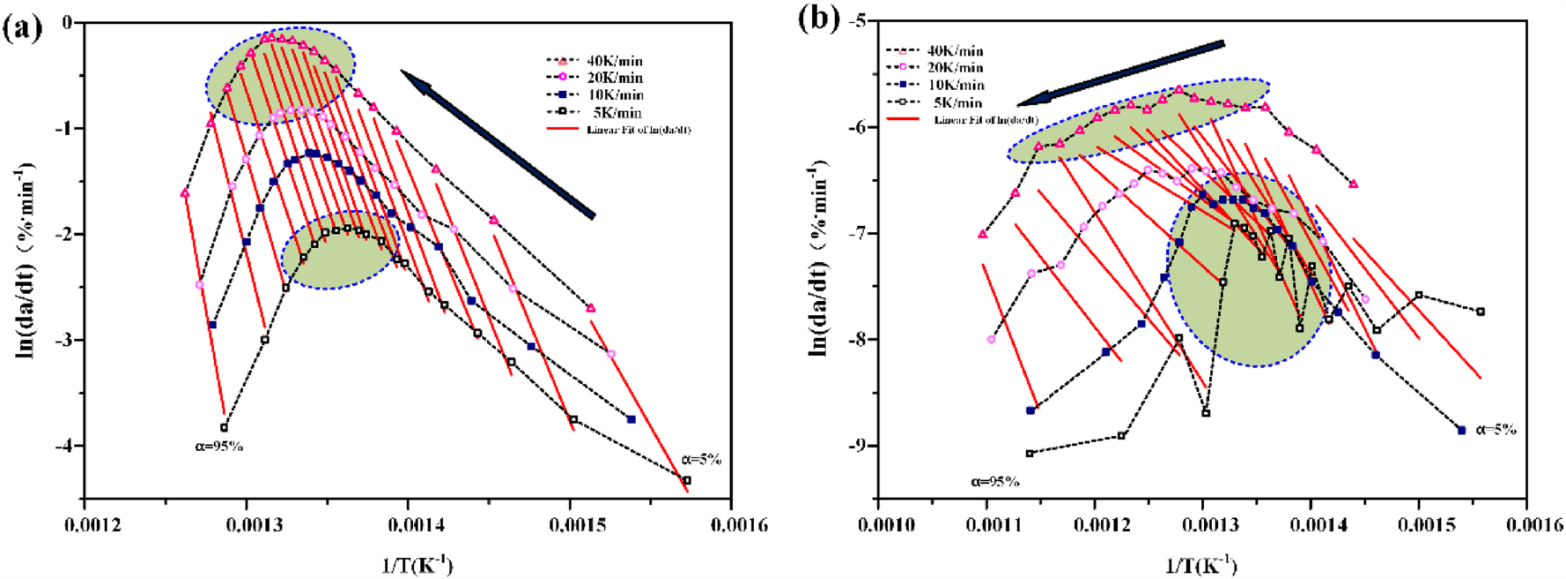
The curve of ln(dα/dt) − (− 1/T) (**a**) atmospheric thermogravimetry (**b**) high pressure thermogravimetry.

Make ln (dα/dt) − (− 1/T) curve, as shown in Fig. 5. The activation energy E_b_ and pre-exponential factor A are calculated according to the slope and intercept.

## Results and discussion

### Shale oil recovery

The goal of oil shale pyrolysis is to obtain shale oil, and shale oil recovery is the most important evaluation parameter. We define the effective recovery of oil shale pyrolysis as the ratio of the quality of shale oil produced by pyrolysis to the oil content of the sample, expressed as a percentage. When high-temperature and high-pressure nitrogen is used as the heat carrier, the experimental results of oil shale pyrolysis are shown in Table 4.

**Table 4:** Product characterization of Huadian oil shale pyrolysis by high-temperature and high-pressure nitrogen.

Number	Temperature/K	Pressure/MPa	Duration/h	Sample/g	Residue/g	Oil/g	Others/g
1	623	7.8	10	100.0	87.5	6.1	6.4
2			20	100.0	86.9	6.7	6.4
3			40	100.1	86.1	7.4	6.6
4			60	100.0	85.3	9.9	4.8
5			80	100.1	84.1	11.7	4.3
6	633		10	100.0	87.9	6.3	5.8
7			20	100.1	86.8	8.8	4.5
8			40	100.0	81.7	9.4	8.9
9			60	100.0	78.4	11.2	10.4
10			80	100.0	76.9	13.3	9.8
11	643		10	100.0	85.3	6.5	8.2
12			20	100.0	81.6	7.4	11
13			40	100.0	77.2	12.3	10.5
14			60	100.0	75.8	14.1	10.1
15			80	100.1	74.6	15.9	9.6
16	653		10	100.0	81.7	7.2	11.1
17			20	100.0	80.9	9.3	9.8
18			40	100.0	79.0	11.1	9.9
19			60	100.0	75.4	15.3	9.3
20			80	100.0	73.4	18.1	8.5
21	663		10	100.0	80.8	8.7	10.5
22			20	100.0	78.8	10.6	10.6
23			40	100.0	76.2	13.2	10.6
24			60	100.0	73.1	18.0	8.9
25			80	100.0	73.5	18.3	8.2
26	673		10	100.0	79.0	11.1	9.9
29			20	100.0	77.8	13.3	8.9
28			40	100.0	75.2	14.7	10.1
29			60	100.0	73.7	18.4	7.9
30			80	100.0	73.3	19.2	7.5

**Figure 6:**
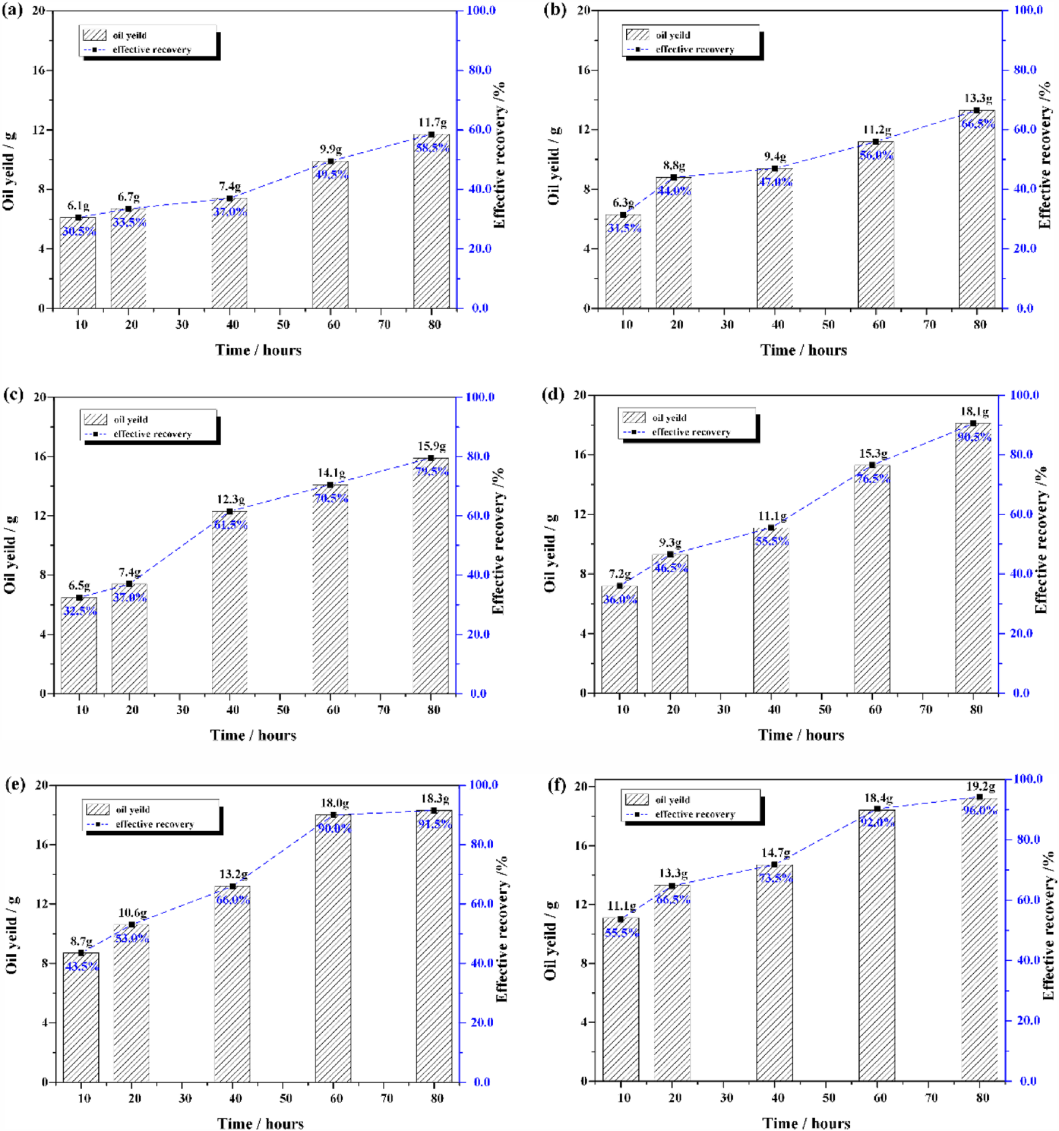
The effective recovery of Huadian oil shale pyrolysis in high-pressure nitrogen atmosphere. (**a**) 623 K; (**b**) 633 K; (**c**) 643 K; (**d**) 653 K; (**e**) 663 K; (**f**) 673 K.

Under high pressure nitrogen atmosphere, temperature and time have different effects on the pyrolysis of Huadian oil shale. When the pyrolysis time is the same, the effective recovery of shale oil will be improved more obviously by increasing the temperature, as shown in Fig. 6.

The effective recovery of shale oil obtained under different atmosphere and temperature attributes vary from 30.5 to 96.0%. When the temperature is 623 K, the pyrolysis time increases from 10 to 80 h, and the effective oil recovery only increases from 30.5 to 58.5%, with a difference of 28.0%. At 673 K, it increased from 55.5 to 96.0%, with a difference of 40.5%. This means that the effect of temperature on shale oil effective recovery is higher than the pyrolysis time. However, when the temperature is higher than 653 K, with the increase of pyrolysis time, although the effective recovery of shale oil also shows an increasing trend, the degree of increase is smaller and smaller.

This is because with the extension of pyrolysis time, the interfacial reaction of oil shale pyrolysis gradually advances to the interior in the closed system, and the higher the temperature is, the faster the interfacial reaction advances. The oil shale is subject to 7.8 MPa confining pressure, so the internal product release process needs to overcome 7.8 MPa pressure, which leads to lower displacement efficiency of heat carrier fluid for internal oil and gas. The oil and gas generated by pyrolysis of oil shale can not be discharged in time, resulting in the intensification of secondary reaction. The macromolecular organic matter that has not been displaced in time under high temperature conditions will break with some active functional groups and generate more gas phase products, thus reducing the increase of effective recovery of shale oil.

### Composition of pyrolysis products

Methane, ethane and propane are the main gaseous products of oil shale pyrolysis under high temperature and high-pressure nitrogen. The components of butane, pentane and their isomers will also appear with the increase of temperature and the extension of time. In addition, it also contains a small amount of hydrogen, ethylene, propylene, etc. The results of normalization calculation of alkane composition and content are shown in Table 5.

**Table 5:** Gas product characterization of oil shale pyrolyzed by high temperature and high pressure nitrogen.

Number	Temperature/K	Pressure/MPa	Duration/h	Methane/%	Ethane/%	Propane/%	Olefin (C_2_–C_4_)/%
1	623	7.8	10	7.57	1.80	1.17	< 0.01(C_3_)
2			20	–	–	–	
3			40	10.80	3.71	2.67	< 0.01(C_3_)
4			60	16.48	5.79	3.89	< 0.01(C_3_)
5			80	18.85	6.12	4.44	< 0.01(C_3_)
6	633		10	8.98	2.73	1.86	< 0.01(C_3_)
7			20	11.78	4.12	2.82	< 0.01(C_3_)
8			40	14.48	6.50	4.60	< 0.01(C_3_)
9			60	16.80	7.80	5.54	< 0.01(C_3_)
10			80	16.94	8.00	5.48	< 0.01(C_3_)
11	643		10	10.60	3.86	2.59	< 0.01(C_3_)
12			20	13.15	6.18	4.45	0.34
13			40	15.72	8.06	5.57	0.23(C_3_ ~ C_4_)
14			60	17.44	8.66	5.98	0.11(C_3_ ~ C_4_)
15			80	18.40	9.89	7.10	0.12(C_3_ ~ C_4_)
16	653		10	13.82	6.44	4.36	0.51(C_3_ ~ C_4_)
17			20	17.13	8.66	5.81	0.30(C_3_ ~ C_4_)
18			40	18.54	9.41	6.31	0.23(C_3_ ~ C_4_)
19			60	21.67	11.18	7.49	0.14(C_3_ ~ C_4_)
20			80	22.97	12.03	8.25	0.06(C_3_ ~ C_4_)
21	663		10	14.45	6.23	4.28	0.38(C_3_ ~ C_4_)
22			20	17.64	8.85	5.83	0.50(C_3_ ~ C_4_)
23			40	20.62	10.73	7.15	0.31(C_3_ ~ C_4_)
24			60	22.47	11.83	8.52	0.22(C_3_ ~ C_4_)
25			80	25.31	13.52	9.46	0.09(C_3_)
26	673		10	19.22	9.84	6.43	0.56(C_3_ ~ C_4_)
29			20	23.20	12.07	7.99	0.45(C_3_ ~ C_4_)
28			40	25.74	13.47	9.23	0.30(C_3_ ~ C_4_)
29			60	28.13	15.21	11.23	0.21(C_3_ ~ C_4_)
30			80	31.26	16.77	13.14	0.11(C_3_ ~ C_4_)

**Figure 7:**
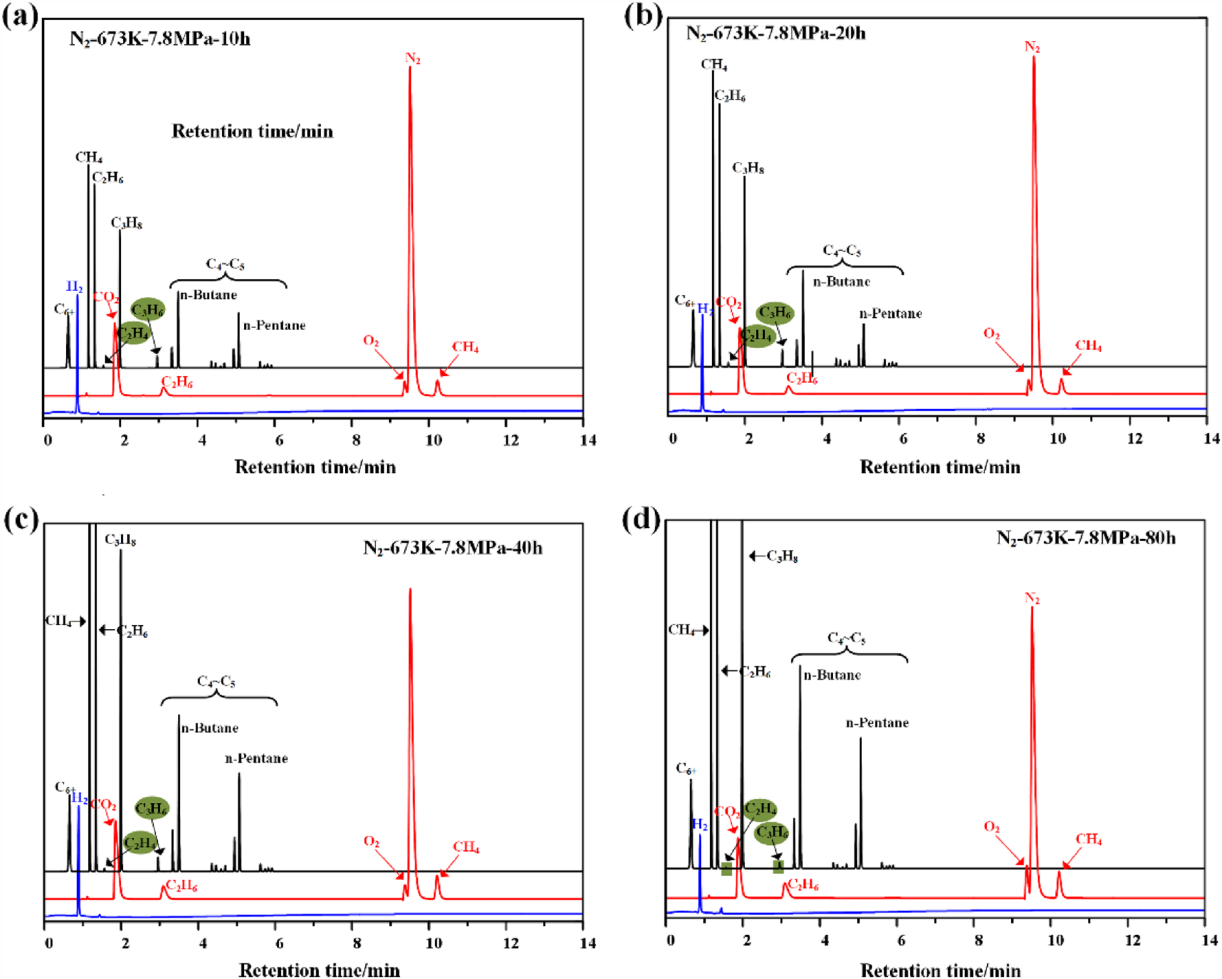
The gas chromatogram of Huadian oil shale pyrolysis gas.

The composition of gas phase products from oil shale pyrolysis does not change, but the content is easy to change, which is caused by the secondary cracking and addition reaction of oil and gas products, as shown in Fig. 7. The secondary cracking becomes more serious with the increase of temperature and the extension of time under high temperature and high-pressure nitrogen atmosphere. Taking 673 K as an example, the pyrolysis time is 10–80 h. After normalization calculation, the content of alkanes continues to rise from 39.41% (including butane, pentane and their isomers) to 58.21%. It also can be verified from the content of C_6+_(steam), methane, ethane, propane and hydrogen in the gas phase products. Of course, it also contains a small amount of olefins. The olefin is mainly composed of propylene, but butene will also appear with the increase of temperature. However, olefins will decrease during long-term pyrolysis at high temperature, indicating that the C=C double bond has broken and the addition reaction with free methyl and hydrogen radicals has taken place. This has promoted the production of ethane, propane, butane and its isomers, pentane and its isomers to a certain extent.

**Figure 8:**
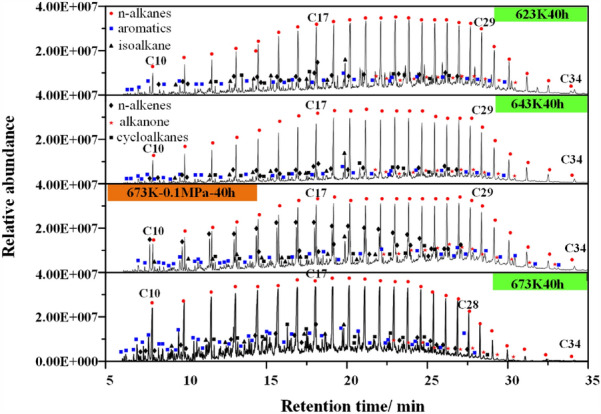
Gas chromatogram of shale oil at different temperatures and pressures.

Shale oil is a typical condensate oil, and its viscosity and phase state change significantly with temperature. The bottom of shale oil collected in each experiment will be brownish paste, while the top will be light yellow transparent. Zhao's research also made similar findings^40^. The composition of shale oil is complex, as shown in Fig. 8. At 623–643 K, it mainly includes normal alkanes, isomeric alkanes, cycloalkanes, olefins, aromatics, branched alkanes, acids and ketones, and the most abundant is normal alkanes.

**Figure 9:**
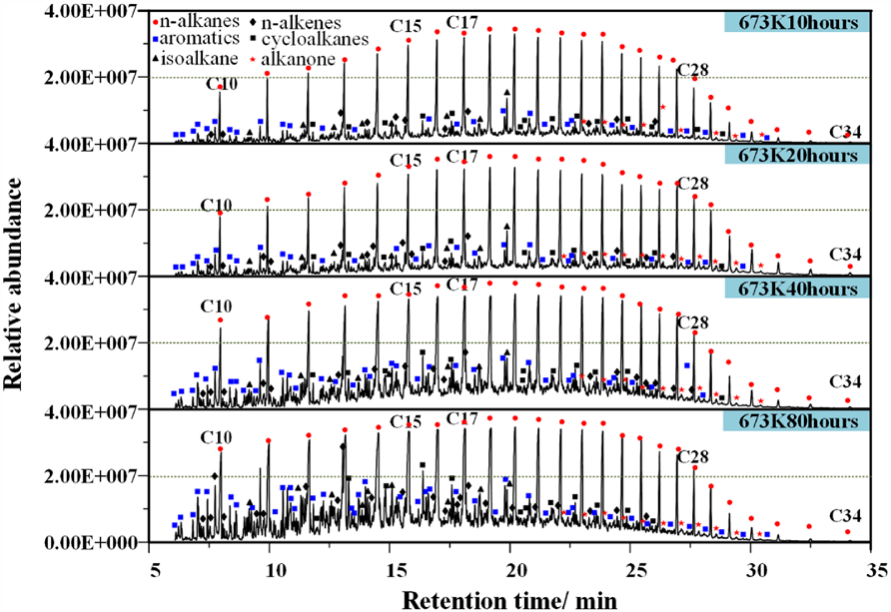
Gas chromatogram of shale oil under different pyrolysis time conditions.

As the temperature gradually rises to 673 K, the pyrolysis time increases from 10 to 80 h, and the content of heteroatomic compounds, cycloalkanes, aromatic hydrocarbons, carboxylic acids and ketones increases, as shown in Fig. 9 and Table 6, which shows that the number of different types of spectral peaks is more in the chromatogram. In addition, pressure also has an important impact on the composition of shale oil, mainly reflected in the content of olefins. During pyrolysis at atmospheric pressure, the characteristic peak of olefins appears with the characteristic peak of alkanes, and the intensity of the characteristic peak of olefins is high. Under high pressure, the synthesis of olefins is inhibited. This is mainly related to the release conditions of products under high pressure, resulting in the secondary addition reaction of olefins in the primary pores of kerogen to generate alkanes.

**Table 6:** The effect of pyrolysis time on product composition at 673 K.

Time (h)	Cycloalkanes (wt%)	Carboxylic acids (wt%)	Aromatic hydrocarbon (wt%)	Ketones (wt%)	Heteroatomic compounds (wt%)
10	2.65	0.11	10.75	0.001	7.59
20	2.91	0.09	12.85	0.003	8.31
40	3.29	0.12	12.64	0.015	10.07
80	3.86	0.15	17.42	0.021	12.37

**Figure 10:**
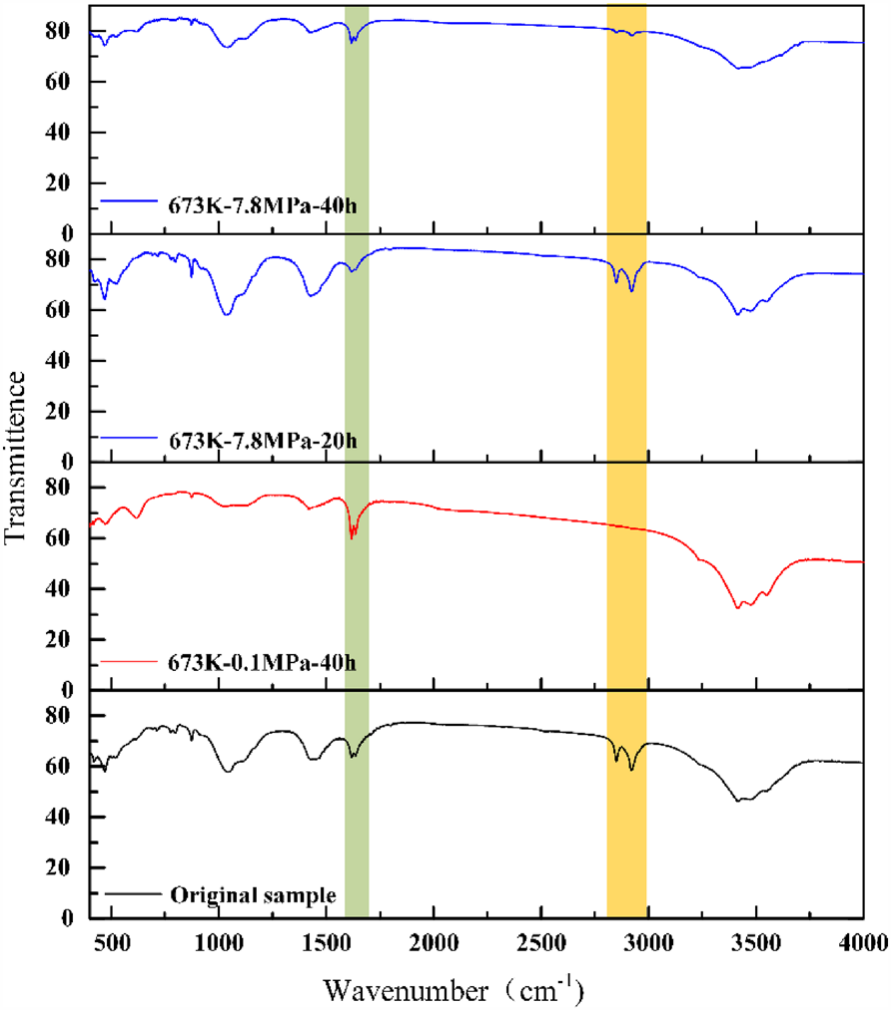
Infrared spectrum of original sample and residue of Huadian oil shale.

Figure 10 shows the infrared characteristic peak spectrum of the Huadian oil shale residue and the original sample under different pressure and pyrolysis time. Among them, 1620 cm^−1^ is the skeleton vibration of aromatic ring, 1710 cm^−1^ is the C–O group stretching vibration. Under different pyrolysis conditions, these two vibrational peaks always exist, indicating that there are aromatic rings and C–O groups in both the original samples and pyrolysis products of Huadian oil shale. The most obvious absorption peaks of organic matter are 2920 cm^−1^and 2871 cm^−1^, which are the characteristic absorption peaks of fatty hydrocarbon, indicating that the main component of organic matter in oil shale is fatty hydrocarbon. Under the pyrolysis condition of 7.8 MPa at 673 K, the absorption peak intensity of fatty hydrocarbons in oil shale residue gradually decreased after the pyrolysis time increased from 20 to 40 h. However, it is still higher than the intensity of the absorption peak of aliphatic hydrocarbon in the oil shale residue sample of 673 K 0.1 MPa pyrolysis for 40 h. This indicates that the process of oil shale pyrolysis under high pressure is slow, and the product release is inhibited.

### Residual pore size and specific surface area distribution

**Figure 11:**
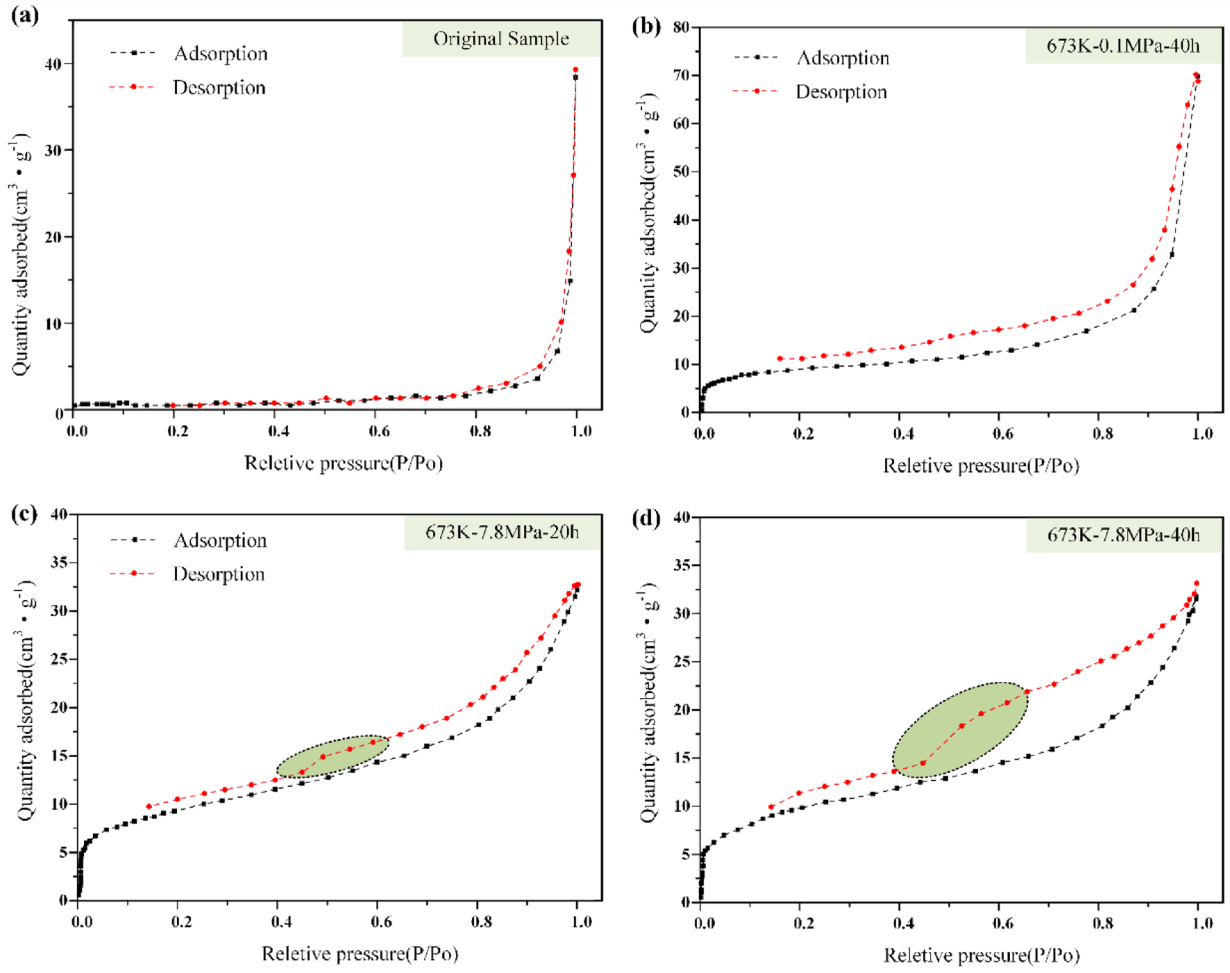
Nitrogen adsorption diagram of original sample and residue of Huadian oil shale.

The pore structure characteristics of shale samples are mainly analyzed by low-temperature nitrogen absorption/desorption experiment, and the sample size is 40–60 mesh. The results are shown in Fig. 11. When P/P_0_ is close to 1, the adsorption and desorption curves of the original oil shale samples overlap (Fig. 11a), while the curves in the semi-coke are separated (Fig. 11b–d). It shows that the pores in the undisturbed oil shale samples are mainly impermeable pores with one end closed, while the pores in the semi-coke are open. By comparing Fig. 11b,d, it can be found that the adsorption line of oil shale residue does not coincide with the desorption line (the relative pressure is greater than 0.2), forming an obvious hysteresis loop. The adsorption isotherm curve and desorption isotherm curve are more open under high pressure, which indicates that the pores in the pyrolysis oil shale residue under high pressure contain more secondary pores. By comparing Fig. 11c,d, it can be found that with the increase of pyrolysis time, the adsorption isotherm curve and desorption isotherm curve are more open. This indicates that the increase of pyrolysis time will also lead to more secondary pores in oil shale. The narrower the hysteresis loop indicates that the sample contains more micropores, and the wider the hysteresis loop indicates that the sample contains more secondary pores.

**Figure 12:**
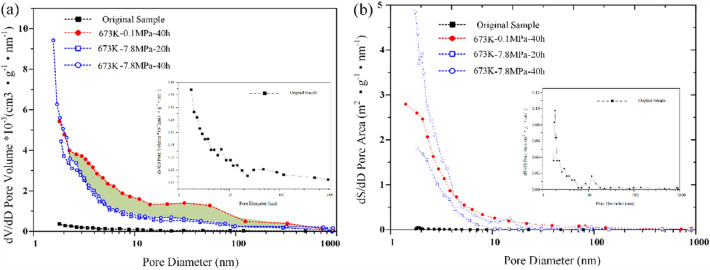
Distribution of pore (**a**) and specific surface area (**b**) of Huadian oil shale.

Figure 12a shows the pore size distribution of oil shale original samples and solid residues under different pyrolysis pressure and time at 673 K. The results show that the undisturbed oil shale sample has a compact structure and only a small amount of micropores. After heating to 673 K, combined with Fig. 11b–d, it can be found that the number, size and structure of pores increase to different degrees, and different pressure and pyrolysis time also have great influence on pore size distribution. In the process of oil shale pyrolysis, the volatilization process will change the physical structure of the residue pores. In addition, volatiles will undergo secondary reactions during the release process, forming numerous secondary pores and changing the pore size distribution. For example, when the same pyrolysis time is 40 h, under the standard atmospheric pressure, the pore development of oil shale residue is more than that under the high-pressure state (Fig. 12a shaded part), especially the number of mesopores and macropores is much higher than that under the high-pressure state. The possible reason is that a large amount of volatile matter precipitates under high pressure, resulting in the collapse of the plastic structure of the particles, which makes some large pores become microporous structure, and the average pore size decreases. This, to some extent, indicates that high pressure inhibits the development of mesopores and macropores in oil shale and promotes the development of micropores^20^.

Compared with the original oil shale sample, the specific surface area of oil shale residue is increased. This is because the process of internal volatiles moving outward during the pyrolysis of oil shale is the process of pore formation. This process increases the surface area and volume of the pore, as shown in Fig. 12b. There are many micropores in oil shale residue under high pressure, so the specific surface area is slightly higher than that under normal pressure. However, when the oil shale is pyrolysis under high pressure, the pressure difference inside and outside the pore increases. Volatile products can be released only when the pressure difference between the internal and external pressure of the pore is greater than the pressure of pore fracture. Therefore, the difficulty of volatile product release increases, which leads to the secondary reaction of some oil and gas products in pores, and forms coke to block pores^41^, so the specific surface area of mesopores and macropores decreases.

### Kinetic behavior of oil shale pyrolysis

**Figure 13:**
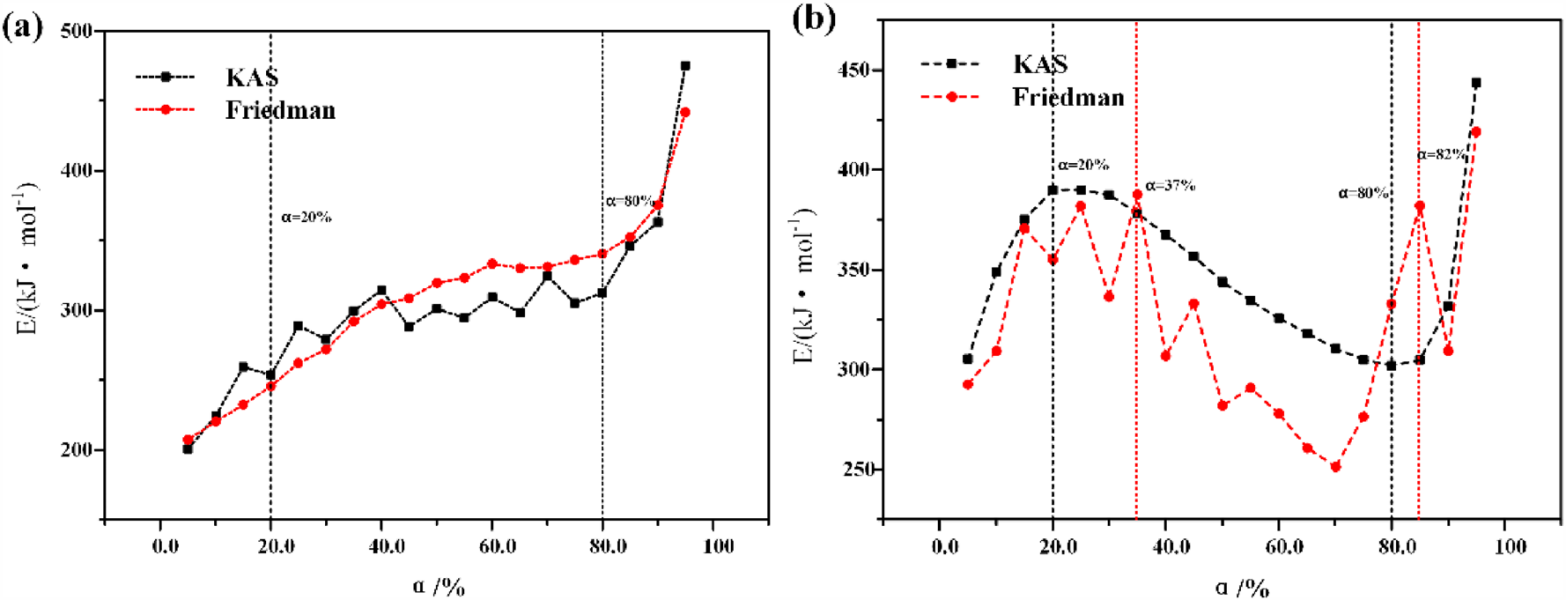
Apparent activation energy of Huadian oil shale pyrolysis under nitrogen atmosphere. (**a**) Standard atmospheric pressure; (**b**) high pressure state.

The activation energy of oil shale pyrolysis under standard atmospheric pressure and high pressure is shown in Fig. 13. It can be seen from Fig. 13a that the activation energy calculated by KAS method and Friedman method shows the same increasing trend with the increase of conversion under standard atmospheric pressure. This is because the pyrolysis process of oil shale particles is gradually advancing from the surface to the interior, and the difficulty of heat and mass transfer is gradually increasing, so the activation energy shows an increasing trend. The average activation energy in the main oil production stage (conversion rate is 20–80%) is about 306.6 kJ/mol. Under high pressure, as shown in Fig. 13b, the activation energy of oil shale pyrolysis shows a trend of increasing first and then decreasing. From 389.9 kJ/mol corresponding to the conversion rate of 20% to 302.1 kJ/mol corresponding to the conversion rate of 80%, the average activation energy in the main oil production stage is still as high as 346.8 kJ/mol. Considering the nitrogen adsorption experiment of oil shale residue comprehensively, we believe that this is related to the release of products that need to overcome higher fracture pressure when oil shale is pyrolysis under high pressure.

**Figure 14:**
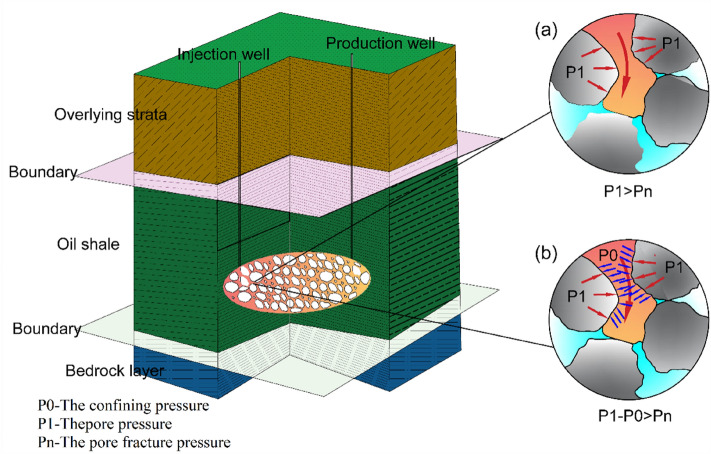
Mechanical analysis of oil and gas product release during oil shale pyrolysis.

As shown in Fig. 14, under standard atmospheric pressure, kerogen undergoes softening, liquefaction and gasification in the pore after being heated, resulting in the gradual increase of pore pressure to P1. When P1 is greater than the pore fracture pressure Pn, oil and gas products can be released and collected. Under the condition of high-pressure pyrolysis, the oil shale pore is under the confining pressure of P0, so only when the pressure P1–P0 in the pore is greater than the fracture pressure Pn, can the oil and gas products be released and collected. The large release of volatile substances will lead to plastic collapse of particle structure. A chain reaction is formed, that is, once a stress weak zone is formed, the stress state of the surrounding pore structure will be changed, and then plastic collapse will occur. This is beneficial to the release of volatile matter to a certain extent, so the activation energy at the main oil production stage shows a downward trend.

## Conclusion

1. The pyrolysis of oil shale is the result of multi-process complex action at high temperature, and is significantly affected by temperature, time and environmental pressure. When the temperature is 623 K, the pyrolysis time increases from 10 to 80 h, and the effective oil recovery only increases from 30.5 to 58.5%. At 673 K, it increased from 55.5 to 96.0%.
2. The secondary cracking becomes more serious with the increase of temperature and the extension of time under high temperature and high-pressure nitrogen atmosphere. At 623–643 K, the composition of gas phase products from oil shale pyrolysis mainly includes normal alkanes. As the temperature gradually increases to 673 K, more chaotic peaks appear in the chromatogram of the pyrolysis products, and the content of heteroatom compounds, cycloalkanes, aromatics, carboxylic acids, and ketones increases.
3. Under the pyrolysis condition of 7.8 MPa at 673 K, the absorption peak intensity of fatty hydrocarbons in oil shale residue gradually decreased after the pyrolysis time increased from 20 to 40 h. However, it is still higher than the intensity of the absorption peak of aliphatic hydrocarbon in the oil shale residue sample of 673 K 0.1 MPa pyrolysis for 40 h. This indicates that the process of oil shale pyrolysis under high pressure is slow, and the product release is inhibited.
4. When the same pyrolysis time is 40 h, under the standard atmospheric pressure, the pore development of oil shale residue is more than that under the high-pressure state, especially the number of mesopores and macropores is much higher than that under the high-pressure state.
5. Under high pressure, the average activation energy of oil shale pyrolysis is 346.8 kJ/mol, greater than that under standard atmospheric pressure of 306.6 kJ/mol. Means the release of products that need to overcome higher fracture pressure when oil shale is pyrolysis under high pressure.

## Data Availability

All data generated or analysed during this study are included in this published article.
